# Assigning factuality values to semantic relations extracted from biomedical research literature

**DOI:** 10.1371/journal.pone.0179926

**Published:** 2017-07-05

**Authors:** Halil Kilicoglu, Graciela Rosemblat, Thomas C. Rindflesch

**Affiliations:** Lister Hill National Center for Biomedical Communications, U.S. National Library of Medicine, Bethesda, MD, 20894, United States of America; Dana-Farber Cancer Institute, UNITED STATES

## Abstract

Biomedical knowledge claims are often expressed as hypotheses, speculations, or opinions, rather than explicit facts (propositions). Much biomedical text mining has focused on extracting propositions from biomedical literature. One such system is SemRep, which extracts propositional content in the form of subject-predicate-object triples called predications. In this study, we investigated the feasibility of assessing the factuality level of SemRep predications to provide more nuanced distinctions between predications for downstream applications. We annotated semantic predications extracted from 500 PubMed abstracts with seven factuality values (fact, probable, possible, doubtful, counterfact, uncommitted, and conditional). We extended a rule-based, compositional approach that uses lexical and syntactic information to predict factuality levels. We compared this approach to a supervised machine learning method that uses a rich feature set based on the annotated corpus. Our results indicate that the compositional approach is more effective than the machine learning method in recognizing the factuality values of predications. The annotated corpus as well as the source code and binaries for factuality assignment are publicly available. We will also incorporate the results of the better performing compositional approach into SemMedDB, a PubMed-scale repository of semantic predications extracted using SemRep.

## Introduction

With the exponential increase in the number of biomedical publications, managing the literature efficiently to support hypothesis generation and discovery has become a daunting task. Text mining from the literature has been proposed to address this challenge [1]. Since the turn of the century, there has been much progress in research focusing on extraction of various kinds of information from the biomedical literature, including various types of named entities (e.g., diseases [2], chemicals [3], genes/proteins [4]) and semantic relations (e.g., gene-disease associations [5], biological events [6], chemical-disease relations [7]).

SemRep [8] is a rule-based, natural language processing system that extracts semantic relations in the form of subject-predicate-object triples (called predications henceforth) from the biomedical research literature. Elements of a predication are drawn from UMLS knowledge sources [9]: predication arguments (subject and object) correspond to UMLS Metathesaurus concepts, and the predicate corresponds to a relation type in an extended version of the UMLS Semantic Network. SemRep is a broad-coverage system in that it extracts relations on a wide range of topics, from clinical medicine (e.g., TREATS, DIAGNOSES, ADMINISTERED_TO) to substance interactions (e.g., STIMULATES, INHIBITS), genetic basis of disease (e.g., CAUSES, PREDISPOSES), and pharmacogenomics (e.g., AUGMENTS, DISRUPTS), as well as some types of static relations (e.g., ISA, PART_OF). Given the input sentence in Example (1a) taken from a PubMed abstract (PMID: 10090351), SemRep generates the predication shown in Example (1b). Mentions corresponding to the predication arguments are underlined and the one corresponding to the predicate is in bold. UMLS concept identifiers (CUIs) of arguments are also provided.

(1)
  (a) *Whether decreased* *VCAM-1* *expression is responsible for the observed* ***reduction*** *in* *microalbuminuria*, *deserves further investigation.*
  (b) C0078056:Vascular Cell Adhesion Molecule-1
    -DISRUPTS-
    C0730345:Microalbuminuria

SemRep relies on the UMLS SPECIALIST Lexicon [10], MedPost part-of-speech tagger [11], and noun phrase chunking, and it is supported by MetaMap [12] for normalizing noun phrases to UMLS Metathesaurus concepts. Entrez Gene [13] serves as a supplementary source to the UMLS Metathesaurus for gene/protein terms. Indicator rules are used to map lexical and syntactic phenomena to predicates. Indicators include lexical categories, such as verbs, nominalizations, and prepositions, and syntactic constructions, such as appositives or modifier-head structure in the simple noun phrase. SemRep underpins the Semantic MEDLINE web application [14] and SemMedDB [15], a PubMed-scale repository of semantic predications, which currently contains more than 85 million predications.

As Example (1) above illustrates, SemRep predications may not fully capture the meaning of the source sentence. It focuses on propositional meaning (the claim that *the reduction in microalbuminuria is due to VCAM-1*) and ignores the semantic layers that a proposition is couched in: that the author of the sentence speculates about the proposition and is *uncommitted* to the factuality of the proposition is not made explicit in the semantic representation. This semantic level is sometimes referred to as *extra-propositional meaning* [16] and its study focuses on phenomena such as uncertainty, negation, hedging, opinions, beliefs, and intentions. Such phenomena are prevalent in biomedical literature, as the scientific method involves hypothesis generation, experimentation, and reasoning on findings to reach, generally tentative, conclusions [17]. Interpreting such phenomena can benefit biomedical text mining applications that rely on semantic relations, by distinguishing facts from tentative statements and allowing inference on the reliability of the underlying scientific claims. For example, Light et al. [18] argued that speculations are more important than established facts for researchers interested in current trends and future directions. It is not difficult to see that the speculative claim in Example (1) above, with its uncommitted status, can form the basis of a new hypothesis and further experiments.

While not as widely studied as more foundational tasks like named entity recognition or relation extraction, in the last decade, there has been some research focusing on extra-propositional meaning in biomedical research literature. Extra-propositional phenomena have been annotated in various corpora. For example, the GENIA event corpus [19] contains biological events from MEDLINE abstracts annotated with their certainty level (certain, probable, doubtful) and assertion status (exist, non-exist). The BioScope corpus [20] consists of abstracts and full-text articles annotated with negation and speculation markers and their scopes. Wilbur et al. [21] proposed a fine-grained annotation scheme with multi-valued qualitative dimensions to characterize scientific sentence fragments, *certainty* (complete uncertainty to complete certainty), *evidence* (from no evidence to explicit evidence), and *polarity* (positive or negative) among them. In a similar vein, Thompson et al. [22] annotated each event in the GENIA event corpus with *meta-knowledge* elements, some of which correspond to extra-propositional aspects: Knowledge Type (Investigation, Observation, Analysis, Method, Fact, Other), Certainty Level (considerable speculation, some speculation, and certainty), and Polarity (negative and positive). Their annotations are more semantically precise as they are applied to events, rather than somewhat arbitrary sentence fragments used by Wilbur et al. [21].

Shared task competitions have provided stimulus in automatic recognition of certain extra-propositional phenomena. BioNLP shared tasks on event extraction [23, 24] and CoNLL 2010 shared task on hedge detection [25] focused on GENIA and BioScope negation/speculation annotations, respectively. Supervised machine learning techniques [6, 26] as well as rule-based methods [27] have been explored in extracting these phenomena and their scopes in these competitions. More generally, Miwa et al. [28] used a machine learning-based approach to assign meta-knowledge categories to events, casting the problem as a classification task and using syntactic (dependency paths), semantic (event structure), and discourse features (sentence location). They also reported state-of-the-art results on the BioNLP shared task corpus. Recently, Kilicoglu et al. [29] proposed a rule-based approach to assign two meta-knowledge categories (Certainty Level and Polarity) to events. The approach extended the Embedding Framework [30], which presents a fine-grained linguistic characterization of extra-propositional meaning and a semantic composition methodology based on lexical information and syntactic dependency parsing to extract such meaning.

The studies that focus on extra-propositional meaning mentioned so far assign discrete values to propositional content (e.g., certainty level or polarity of an event). While these values can be useful for downstream applications, a potentially more useful notion is *factuality*, which can be conceived as a continuum that ranges from factual to counter-factual with degrees of uncertainty in between. In computational linguistics, factuality is often modeled as the interaction of *epistemic modality* (or certainty) and *polarity* [31]. By making the interaction of these dimensions explicit, factuality values allow us to compositionally model the effect of fragments like *unable to demonstrate* and *can* on the factuality of the proposition that *DA prevents acute renal failure* in the sentence *Studies have been unable to demonstrate that DA can prevent acute renal failure*. Furthermore, we can draw inferences about ordering of different propositions by their factual status more readily (i.e., is proposition X more likely than proposition Y?) or to distinguish how a given proposition is presented in different contexts (i.e., is claim X presented as a fact in publication A and as unlikely in publication B?). Such inferences can enable tasks like tracking of scientific claims, among other possible uses. Kilicoglu et al. [29] demonstrated how biomedical event factuality can be inferred from the results of semantic composition; however, their evaluation mainly focused on *certainty* and *polarity* categories, in the absence of a corpus annotated with factuality.

In this study, we focus on the factuality of SemRep predications. We present an annotated corpus in which we annotated SemRep predications extracted from 500 PubMed abstracts with their factuality values. We extend our earlier work [29] to compositionally predict factuality values. With the availability of an annotated corpus, we also experiment with a supervised machine learning approach based on a rich feature set. Our results indicate that the rule-based approach outperforms the machine learning approach in predicting factuality of predications. The rule-based factuality assessment of SemRep predications will be incorporated into SemMedDB, allowing for a more fine-grained input for advanced data mining and literature-based discovery applications that rely on this repository.

## Methods

In this section, we first discuss our corpus and the annotation study. We then describe two approaches to factuality assessment of SemRep predications, the first a rule-based, compositional approach and the second a machine learning-based method. We conclude this section by briefly discussing the evaluation methodology.

### Corpus and annotation

For training and testing, we used a corpus of 500 PubMed abstracts. These were randomly selected from a larger corpus of approximately 45,000 abstracts used in another study (as of yet unpublished). The selection criteria for the abstracts in the larger corpus was that SemRep extracted at least one predication from them, in which the object argument was one of a small number of disorders, including Alzheimer’s disease, asthma, myocardial infarction, obesity, and Parkinson disease, and the predicate type was one of CAUSES, PREVENTS, PREDISPOSES, and TREATS.

**Fig 1:**
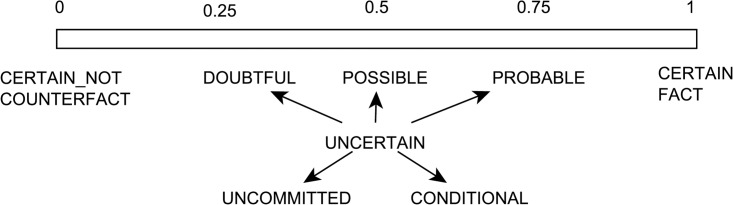
The factuality scale with proposed factuality values.

We annotated SemRep predications extracted from these abstracts with seven discrete factuality levels: fact, probable, possible, doubtful, counterfact, uncommitted, and conditional. The motivation in selecting these values was to be as fine-grained as possible while also maintaining annotation feasibility. We also aimed to be consistent with existing categorizations and were inspired, in particular, by several other characterizations of factuality and certainty levels [19, 31, 32]. Among these seven values, the first five can be modeled on a factuality scale, where fact and counterfact represent the endpoints and probable, possible, and doubtful represent the intermediate values from more to less factual. uncommitted and conditional values, on the other hand, are inspired by Szarvas et al. [32] and Saurí and Pustejovsky [31] and aim to model predications which are underspecified with regards to their factuality. uncommitted predications are those propositions whose factuality value is unknown, while conditional predications are those whose factuality value depends on the factuality of another predication. Examples for each type are given in Table 1 and the factuality scale is illustrated in Fig 1.

**Table 1:** Examples of SemRep predications with their factuality values.

Sentence	Predication	Factuality
*Nifedipine* ***increased*** *renal blood flow*, *both in salt-sensitive and in salt-resistant individuals …*	Nifedipine-AUGMENTS-Renal Blood Flow	fact
*Our findings support the hypothesis that* *tamoxifen* *increases the* ***risk*** *of* *endometrial carcinoma* *and premalignant changes …*	Tamoxifen-PREDISPOSES-Endometrial Carcinoma	probable
*Women identified as being at high risk for* *breast cancer* *as determined by these hormone levels may* ***benefit*** *from* *antiestrogen* *treatment for primary prevention.*	Estrogen Antagonists-TREATS-Malignant neoplasm of cancer	possible
*These results obtained with a limited number of patients do not support any clinical efficacy of regular* ***treatment*** *with an oral* *antileukotriene* *in* *seasonal allergic rhinitis* *…* …	Leukotriene Antagonists-TREATS-Hay fever	doubtful
*Losartan*, *an angiotensin II receptor antagonist*, *does not* ***produce*** *cough* *which is classically seen with ACE inhibitors.*	Losartan-CAUSES-Coughing	counterfact
*Plasmapheresis* ***for*** *collagen diseases.*	Plasmapheresis-TREATS-Collagen Diseases	uncommitted
*Cyclic AMP* *was found to either inhibit or markedly* ***increase*** *CD40L* *expression dependent upon the mechanisms of T cell activation.*	Cyclic AMP-STIMULATES-CD40 Ligand	conditional

For each predication extracted by SemRep, the annotators performed the following two steps:

- If the extracted predication is a false positive, mark it as such (i.e., no further factuality annotation is needed).
- Otherwise (true positive), mark the factuality level of the predication using one of the seven factuality values.

Initially, all predications were automatically marked as fact. No specific annotation guidelines were developed. Instead, the annotators read the relevant papers on factuality categorization (in particular, Saurí and Pustejovsky [31] and Szarvas et al. [32]), discussed their understanding, and developed the 7-level categorization in the light of SemRep predications. In the first phase of the annotation, 50 abstracts were double-annotated by two of the authors (HK, GR). The annotations were then compared and reconciled and inter-annotator agreement was calculated. Reconciliation helped to clarify some of the issues with factuality annotation of SemRep predications. Following the reconciliation, one of the authors (GR) annotated the rest of the corpus (450 abstracts). The corpus was then split into two for this study: 300 abstracts were used for training and 200 abstracts were used for testing. The annotation was performed using the *brat* annotation tool [33]. To measure inter-annotator agreement, we calculated Cohen’s kappa (*κ*) [34] coefficient, which takes into account chance agreement, as well as the F_1_ score when one set of annotations is taken as the gold standard, which does not consider chance agreement. The annotated corpus is publicly available at https://skr3.nlm.nih.gov/Factuality/ (a UMLS license is required).

### Compositional factuality assessment

**Fig 2:**
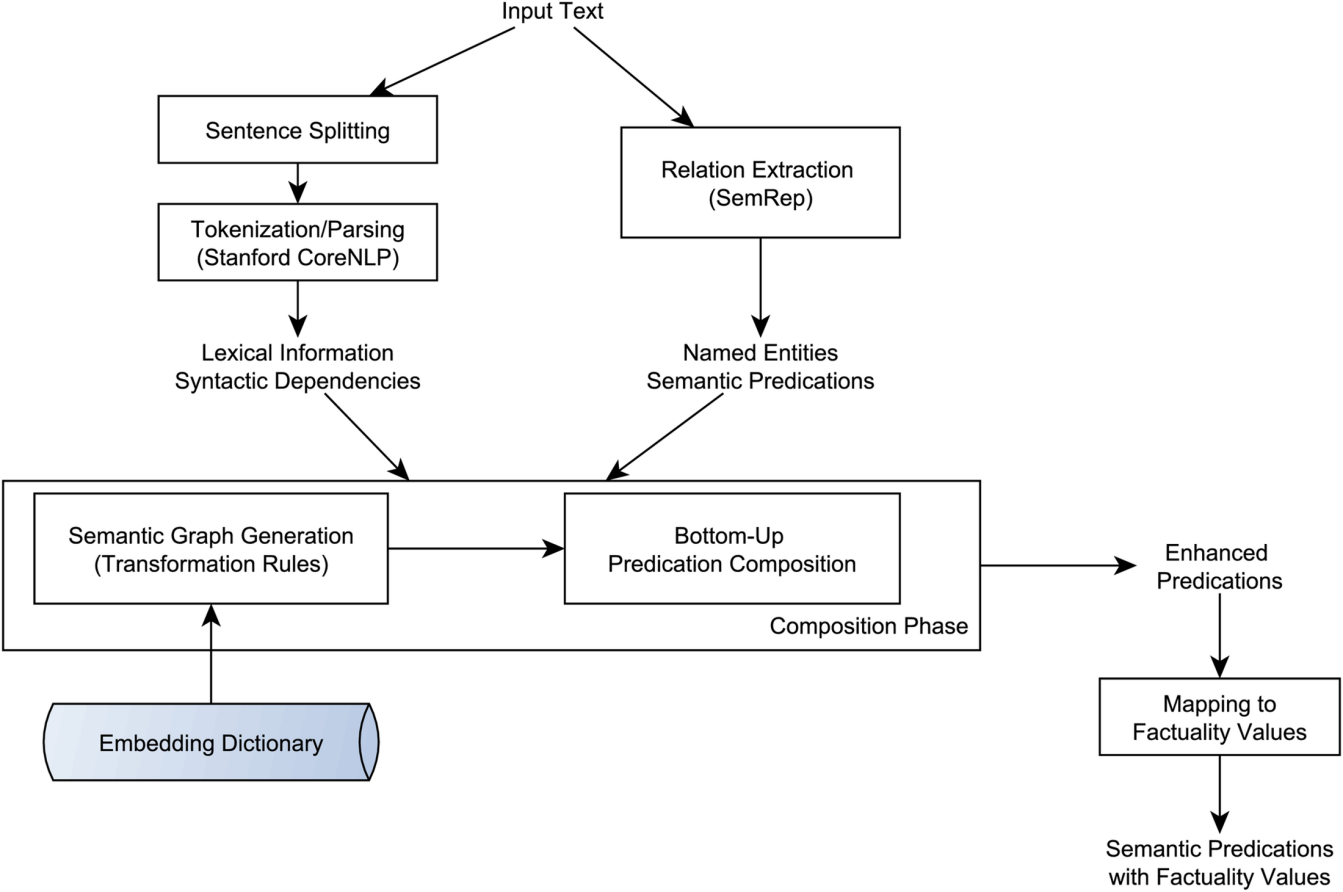
Workflow diagram of the method for compositional factuality assessment of SemRep predications.

We experimented with two approaches to assess factuality of SemRep predications. The first approach is an enhancement of the compositional approach reported in earlier work [29], in which the method was applied to assess the factuality of biological events in the GENIA event corpus [19]. In this paper, we briefly describe the core of this approach and how it was enhanced for SemRep predications. A more detailed description can be found in Kilicoglu et al. [29]. The high-level workflow in this approach is illustrated in Fig 2.

The compositional approach is based on the Embedding Framework [30], which views *extra-propositional meaning* as a domain-independent semantic layer and focuses on modeling it in a unified manner. This framework can be viewed as being complementary to SemRep, which explicitly focuses on propositional meaning, and consists of the following components:

- An enhanced predication representation
- A domain-independent categorization of embedding phenomena
- A dictionary of embedding predicates
- A predication composition procedure that uses syntactic dependency relations and the components above to extract enhanced predications

The framework can incorporate conceptual and propositional information in the form of named entities and relations extracted by third-party tools, as long as the textual mentions of relevant terms and predicates are provided. SemRep, with its explicit marking of entities and predicates, therefore, readily provides the semantic information that can be integrated into the framework. Enhanced predications generated by the predication composition procedure can be used to address various practical tasks. Factuality assessment is one such task and is achieved by rules that map each enhanced predication to a distinct factuality level.

We illustrate the components of the framework on an example, shown in Table 2. The sentence under consideration is given in row (1), while the named entities and the predication provided by SemRep are in rows (2) and (3), respectively. The enhanced predications generated by the framework are illustrated in row (4). Note that the named entities in row (2) appear with their term identifiers in row (4) for readability (*t*_1_, *t*_6_, etc.). In the Embedding Framework, a predication is formally defined as:

$$\begin{array}{c} Pr≔[P,S,MV_{Sc},Arg_{1..n}],n>=1 \end{array}$$

where *P* is a typed predicate, *S* is the source of the predication (attribution), and *MV_*Sc*_* is its *scalar modality value*. *Arg*_1..*n*_ represent the logical arguments of the predication (shown in *object*, *subject*, and *adjunct* order), some of which can be other predications (i.e., predications can be nested).

**Table 2:** Composition example for a sentence in PMID 10652588.

(1)	*These* *results* *suggest that* *Ibuprofen* *may have* *potential* *in the* *chemoprevention* *and* *treatment* *of* *breast cancer.*
(2)	*results(t*_1_*)* ⇒ C1274040:result (Functional Concept) *Ibuprofen(t*_2_*)* ⇒ C0020740:Ibuprofen (Organic Chemical, Pharmacologic Substance) *potential(t*_3_*)* ⇒ C0237399:Potential (Qualitative Concept) *chemoprevention(t*_4_*)* ⇒ C0282515:Chemoprevention (Therapeutic or Preventive Procedure) *treatment(t*_5_*)* ⇒ C0087111:Therapeutic procedure (Therapeutic or Preventive Procedure) *breast_cancer(t*_6_*)* ⇒ C0006142:Malignant neoplasm of breast (Neoplastic Process)
(3)	C0020740:Ibuprofen-TREATS-C0006142:Malignant neoplasm of breast
(4)	*treatment*:treats *(e*_1_,*t*_1_,*0.5*_*epistemic*_,*t*_6_, *t*_2_*)* *may*:speculative *(em*_10_,*t*_1_,*0.75*_*epistemic*_,*e*_1_*)* *suggest*:deductive *(em*_11_,*WR*,*1.0*_*epistemic*_,*em*_10_,*t*_1_*)*

In row (2), UMLS Metathesaurus concepts corresponding to entities are represented as *CUI: Preferred Name (Semantic Types)* tuples.

In the example in Table 2, the SemRep predication in (3) is incorporated into the framework as *e*_1_ (row (4)). Its source *S* has been identified as *t_1_*, the term corresponding to the mention *results*. The predication is placed on the *epistemic scale*, with the modality value of 0.5. The framework also generates two new embedding predications (*em*_10_, *em*_11_), indicated by *may* and *suggest*, typed as speculative and deductive predications, respectively. Note that these two predications are nested: *e_1_* is embedded by *em*_10_, which in turn is embedded by *em*_11_. *e*_1_ and *em*_10_ are also said to be within the scope of *em*_11_. The speculative predication (*em*_10_) has the same source as the TREATS predication (*e*_1_), while the deductive predication is attributed to the author of the document (wr). Both embedding predications are also placed on the epistemic scale (or factuality scale). In our model, as shown in Fig 1, an epistemic scale value of 1 corresponds to a fact while the value of 0 corresponds to a counterfact, and the intermediate values can be paraphrased as (in increasing order) doubtful, possible, and probable. So, in essence, the enhanced predications in row (4) of Table 2 can be paraphrased as follows:

- *e*_1_: The treatment of breast cancer by Ibuprofen is possible according to *these results*.
- *em*_10_: The possibility that Ibuprofen treats breast cancer is probable, according to *these results*.
- *em*_11_: That *these results* suggest the possibility that Ibuprofen may treat breast cancer is a fact according to the author.

**Fig 3:**
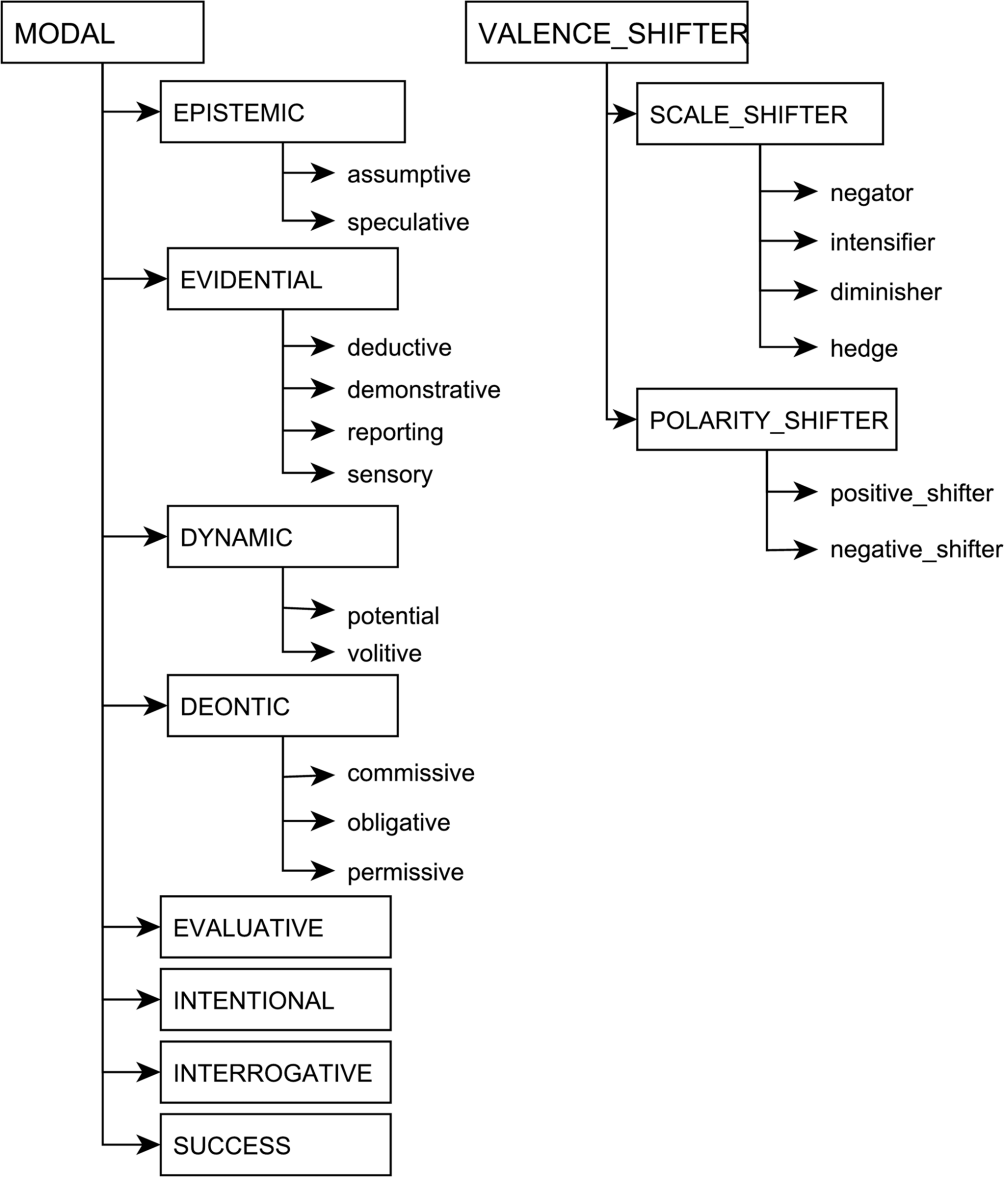
Embedding categorization.

A domain-independent embedding categorization and a dictionary in which embedding predicates, such as *may* and *suggest*, are mapped to these categories, underpin the composition of these predications. Four main types of extra-propositional predicates are distinguished in the categorization: modal, relational, valence_shifter and propositional, each of which is subdivided into subcategories. For factuality assessment, modal predicates as well as scale_shifter predicates (a subcategory of valence_shifter) are the most relevant. These two categories are illustrated in Fig 3. Without elaborating on individual subcategories, we point out that predicates belonging to modal subcategories are associated with modality scales (e.g., *epistemic scale*, *deontic scale*) which are modeled with the [0,1] range. The predications within the scope of these predicates are placed on the relevant scale, where a value of 1 indicates strongest positive association with the scale and 0 indicates negative association. Conversely, scale_shifter predicates do not introduce modality scales but trigger a scalar shift of the embedded predication on the associated scale.

**Fig 4:**
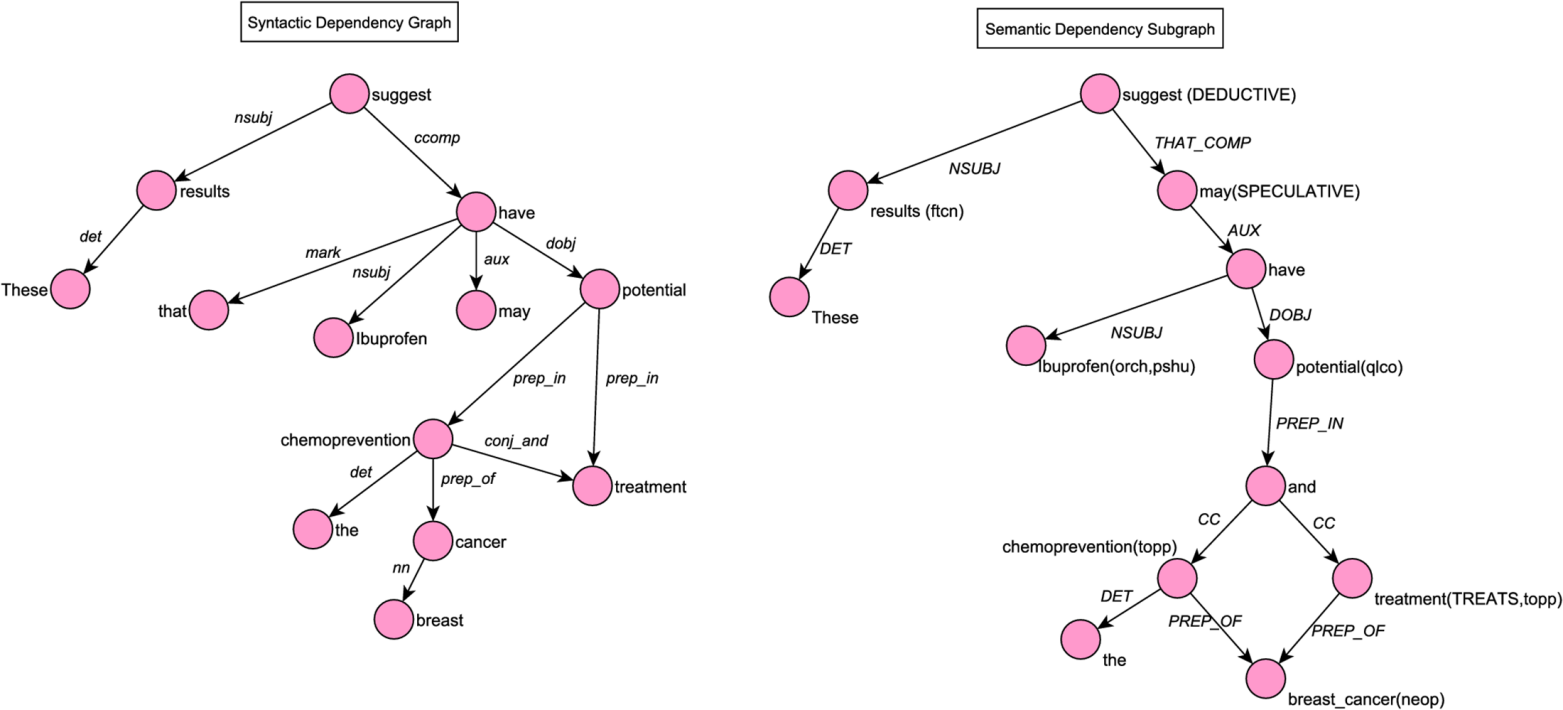
Syntactic dependency graph of the sentence *These results suggest that Ibuprofen may have potential in the chemoprevention and treatment of breast cancer*. and its corresponding semantic dependency subgraph.

Predication composition also relies on lexical information (tokens, lemmas, part-of-speech) and syntactic dependencies provided by the Stanford CoreNLP toolkit [35]. The collapsed format of dependency relations is used. In the first step of composition, syntactic dependency graphs of abstract sentences are transformed into a semantically enriched, directed, acyclic semantic document graph through a series of dependency transformation rules. Each node of the semantic graph corresponds to a textual unit of the document, potentially associated with some semantics, and the direction of edges between them reflects the *semantic dependency* between its endpoints, rather than a syntactic dependency. The syntactic dependency graph of the sentence in Table 2 and the corresponding semantic subgraph are shown in Fig 4. In generating this semantic dependency subgraph, two transformation rules have been used (Verb Complex Transformation and Coordination Transformation). The former rule applies to the nodes associated with the verbs *suggest* and *have*, while the latter applies to the dependency relation *conj_and* between the nodes *chemoprevention* and *treatment* (see [30] for more details on the transformation rules).

The generation of the semantic graph is followed by bottom-up traversal of the graph for predication composition. Two procedures in predication composition that play roles in factuality assessment are:

- *Argument identification* is the process of determining the logical arguments of a predication, using rules based on the lexical/syntactic properties of its predicate defined in the dictionary.
- *Scalar modality value composition* determines the relevant scale and modality value on this scale for predications in the scope of a modal or scale_shifter predicate.

Each predication is initially placed on the epistemic scale and assigned the modality value of 1 (i.e., fact). Scalar modality value composition then modifies this value as appropriate during traversal of the graph. Information about the predicates that is needed for the composition procedures is compiled in the hand-crafted embedding dictionary, which currently contains 548 modal and 97 scale_shifter predicates. The dictionary entry for the modal auxiliary predicate *may* is given in Table 3. It is defined as having two modal senses: speculative and permissive (i.e., it is ambiguous). According to this entry, when it indicates speculative meaning (Sense.1), the predication in its scope is placed on the epistemic scale (since speculative is a subcategory of epistemic) and assigned an initial value of 0.5. The semantic dependency type *AUX* is expected between *may* and the predicate corresponding to the predication that it embeds. This entry licenses the TREATS predication indicated by *treatment* in Fig 4 as being in the scope of the speculative *may*, since there is a semantic dependency with type *AUX* on the path between the corresponding nodes. Though not associated with a predication, the verb *have* is also taken as the logical object argument of the speculative *may* (*argument identification*, due to same semantic dependency.

**Table 3:** Dictionary entry for the modal auxiliary *may*.

Lemma POS	*may* *MD* (modal)	
Sense.1	Category	speculative
	Prior scalar modality value	0.5
	Semantic dependency types	*AUX*
Sense.2	Category	permissive
	Prior scalar modality value	0.6
	Semantic dependency types	*AUX*

#### Enhancing the compositional approach

To use the compositional approach with SemRep predications, we make several enhancements in the composition procedure. First, we prune the embedding predicate annotations of the system that are subsumed by or overlap with named entities or predicates identified by SemRep, in order to avoid using them for factuality assessment and to give precedence to the semantics generated by SemRep. For example, *potential* in the sentence in Fig 4 is mapped to a UMLS concept and it is also an embedding predicate belonging to the potential subcategory of the dynamic modal category. The latter sense is, therefore, pruned from the semantic graph, as illustrated in Fig 4.

Second, the handling of negated predication arguments is enhanced. Negation triggers, such as *no* or *neither*, can affect the factuality of the predication itself when they modify a predication argument, since they can have wide scope [36]. In the previous study [29], while we were able to achieve state-of-the-art performance for certainty, our results for polarity (negation) lagged behind. Our error analysis revealed that the system did not address negation of predication arguments well. We previously assumed that, for such wide scope interpretation, the predicate corresponding to a predication should directly dominate the negation trigger, which in turn should directly dominate the predication argument in the semantic graph. This often does not hold, particularly with SemRep predications, since SemRep uses an underspecified approach in semantic interpretation and the syntactic/semantic dependencies between the predicate and the arguments are often not captured neatly in the semantic graph. To improve the handling of negated arguments, we relax this assumption and stipulate that an argument be negated if there is an appropriate negation trigger node anywhere on the dependency path between the predicate and the argument nodes, making the predication negated, as well. This enhancement allows us to capture that the INHIBITS predication in Example (2) is a counterfact.

(2)
  - *CC1069, but not the parent drug* *thalidomide*, ***inhibited*** *in vitro production of* *TNF-alpha* *…*
  - Thalidomide-INHIBITS-TNF-alpha

Third, we add a semantic graph path constraint between the embedding predicate and the predicate indicating the SemRep predication. This constraint applies when the embedding predicate is an adverb or a modal auxiliary, and stipulates that for the SemRep predication to be in the scope of the embedding predicate, no verbal node can be on the path between the embedding predicate and the SemRep predicate, unless the verbal node corresponds to a light verb, such as *associate* or *have*. In Example (3) below, this constraint correctly prevents the predicate *effect* from being under the scope of the negation trigger *not*, since two verbs, *designed* and *investigate*, appear on the semantic graph path (relevant graph dependencies are illustrated below). Therefore, the predication shown is a fact, rather than a counterfact.

(3)
  - *The patients were drawn from a larger placebo-controlled, double-blind, randomized trial, which was not originally designed to investigate the* ***effect*** *of* *pramipexole* *on* *tremor.*
  - Pramipexole-AFFECTS-Tremor
  - NEG(not,designed)
    XCOMP(designed,investigate)
    DOBJ(investigate,effect)

#### Mapping predication elements to factuality values

Once the enhanced predications are composed, we use simple rules to assign factuality levels to them. In our previous work, these rules were essentially based on scalar modality values associated with predications. In the current study, based on the analysis of the training examples, these were slightly modified, with new rules added for uncommitted factuality value, since non-commitment had not been addressed in earlier work. In addition, we developed several rules based on the predicate or indicator type of the SemRep predication, since we found that some predicate or indicator types predominantly indicate factual predications. These rules are as follows:

- All ISA predications are assigned the factuality value of fact.
- All predications indicated by modifier-head constructions or prepositional predicates are assigned the value of fact.
- All inferred predications (INFER) inherit the factuality value of their non-inferred counterpart.

The first two rules aim to address predominantly factual static relations (rather than events).

The modified, scalar modality value-based rules are shown in Table 4. We did not address the conditional factuality value, since only a single instance was annotated in the corpus.

**Table 4:** Mapping scalar modality values to factuality levels.

Condition	Factuality value
MV_*epistemic*_ = 1	fact
MV_*epistemic*_ >= 0.65 AND MV_*epistemic*_ < 1	probable
MV_*epistemic*_ > 0.25 AND MV_*epistemic*_ < 0.65	possible
MV_*epistemic*_ > 0 AND MV_*epistemic*_ <= 0.25	doubtful
MV_*success*_ = 1	fact
MV_*success*_ >= 0.65 AND MV_*success*_ < 1	probable
MV_*success*_ > 0.25 AND MV_*success*_ < 0.65	possible
MV_*success*_ > 0 AND MV_*success*_ <= 0.25	doubtful
MV_*potential*_ > 0	probable
MV_*epistemic*_ = 0 OR MV_*potential*_ = 0 OR MV_*success*_ = 0	counterfact
MV_*interrogative*_ = 1	uncommitted
MV_*deontic*_ > 0.65 AND MV_*deontic*_ < 1	uncommitted

Note that only the enhanced predications corresponding to SemRep predications are mapped. All other predications are pruned at this step, since their factuality values are not of interest for this study.

### Factuality assessment with supervised machine learning

As an alternative to the compositional approach, we experimented with a machine learning method. We cast factuality prediction as a multi-label classification task. LIBLINEAR implementation of linear SVM [37] was used as the learning algorithm. The regularization parameter *C* was set to 2, using grid search.

We experimented with two sets of features. The first set consists of features used by Miwa et al. [28] for classification of meta-knowledge dimensions. We used this set of features, because two meta-knowledge dimensions (Certainty Level and Polarity) are relevant to factuality and they report state-of-the-art results on classification of these dimensions. The features they used are classified as follows:

- *Shortest path features* are computed over the syntactic dependency paths between meta-knowledge clues and event participants (triggers and arguments) and include features such as the shortest path length, n-gram features (n = 1,2,3,4) over the shortest path vertices (tokens) and edges (dependency labels). As meta-knowledge clues, we used the predicates in the embedding dictionary. Event participants are analogous to predication elements (predicate and subject-object pair).
- *Trigger features* represent the context around the event trigger (predicate) and are computed over 2-step dependency paths from the trigger. These features include n-gram features (n = 2,3) over the tokens in the path as well as bigram features over the dependency labels and n-gram features (n = 2,3,4) over both token and dependency labels.
- *Event trigger-argument pair features* are n-gram features (n = 1,2,3,4) within a window of three tokens before the first token and three tokens after the last token in the trigger-argument pair.
- *Sentence features* are the absolute position (first, second, etc.) and the relative position of the predication sentence in the abstract.

While we aimed to replicate the experimental setting of Miwa et al. [28], this was not entirely feasible since they used different tools for linguistic analysis and based their classification on their event extraction system, EventMine [38]. Furthermore, their meta-knowledge clue list was not available to us. Therefore, our implementation is a rough approximation of their method.

The second set of features include a subset of the features above as well as several additional features, some incorporating SemRep-specific information and others that were expected to be helpful for the classification task. The features that are shared with those of Miwa et al. [28] are:

- *Shortest path length features* between an embedding predicate and the predication elements (predicate, subject, and object). Note, however, that these features are computed over the semantic dependency graph instead of the syntactic dependency graph.
- Trigger-argument pair unigram and bigram features
- Sentence relative position feature

The additional features used in the second set are the following:

- *Dominating embedding triggers feature* indicates the list of embedding triggers that dominate the predicate in the semantic dependency graph.
- Feature indicating whether the predication sentence is in the title of the abstract.
- Predicate type feature (TREATS, ISA, PROCESS_OF, etc.)
- Indicator type feature (whether it is a verb, noun, preposition, etc.)
- *The scalar modality value feature* depends on the semantic composition procedure outlined above. The scalar modality value associated with the enhanced predication is discretized, such that the modality scale is divided into 5 bins. The mapping of scalar values to bins is as follows: {1.0 → 5, 0.65-0.99 → 4, 0.26-0.64 → 3, 0.01-0.25 → 2, 0.0 → 1}. For example, if the predication has the scalar modality value of 0.9_*epistemic*_, this is discretized as EPISTEMIC_4.

### Evaluation

We assessed our methodology on the annotated factuality corpus. We used a simple majority baseline, which indicates that all predications have the factuality level of fact, essentially what SemRep currently assumes. In one experiment, we used the system we reported earlier for factuality assessment of GENIA events [29] as-is. That system generated Certainty Level (L3, L2, L1) and Polarity (Positive, Negative) annotations. L3 corresponds to a fact, L2 indicates high confidence (slight speculation) and L1 indicates considerable speculation. We simply mapped these values to our categorization as shown in Table 5. This earlier system did not address the factuality value uncommitted.

**Table 5:** Mapping Certainty Level and Polarity to factuality values.

Certainty Level	Polarity	Factuality
L3	Positive	fact
L2	Positive	probable
L1	Positive	possible
L1 OR L2	Negative	doubtful
L3	Negative	counterfact

We also evaluated the enhanced compositional factuality assessment method, as well as the supervised machine learning method with two sets of features. We used precision, recall, and F_1_ score as evaluation metrics for individual factuality levels, and accuracy as the metric for overall factuality assessment.

## Results and discussion

In this section, we present the results of the annotation study as well as those of rule-based and machine-learning approaches to factuality prediction. We conclude the section by providing an error analysis and discussing some negative results.

### Annotation study and inter-annotator agreement

The statistics regarding the corpus are given in Table 6. In terms of inter-annotator agreement, Cohen’s kappa (*κ*) value was 0.75, which is considered *substantial agreement*. Inter-annotator agreement based on F_1_ score, on the other hand, was 0.91.

**Table 6:** SemRep factuality corpus characteristics.

	# Training (%)	# Testing (%)	# Total (%)
Abstracts	300	200	500
SemRep predications	4,431	2,960	7,391
True positive SemRep predications	3,149 (71.1)	2,179 (73.6)	5,328 (72.1)
fact	2,754 (87.5)	1,958 (89.9)	4,713 (88.4)
probable	143 (4.5)	67 (3.0)	210 (4.0)
possible	66 (2.1)	61 (2.8)	127 (2.4)
doubtful	8 (0.3)	6 (0.3)	14 (0.3)
counterfact	57 (1.8)	35 (1.6)	92 (1.7)
uncommitted	120 (3.8)	52 (2.4)	172 (3.2)
conditional	1 (0.0)	0 (0.0)	1 (0.0)

As the statistics in Table 6 show, the corpus is dominated by factual predications. This is largely consistent with the distribution in similar corpora. For example, in Thompson et al. [22], factual events (indicated by L3 Certainty Level and Positive polarity) account for 86.5% of all events. We attribute the difference in our distribution (88.4% vs. 86.5%) to the fact that SemRep attempts to address not only events but also some static relations (ISA, PART_OF), which are overwhelmingly factual. While we obtained substantial inter-annotator agreement without annotation guidelines, it is likely that they could have been beneficial in obtaining even better agreement, as reported in Thompson et al. [22] (0.93 *κ* for Polarity and 0.86 for Certainty Level).

### Factuality assessment

Evaluation results for factuality assessment are presented in Table 7. These results show that a simple majority baseline, which is clearly not very useful, already yields an accuracy of 89.9%. Using the earlier rule-based system [29] as-is with the simple mappings in Table 5, the results are in fact poorer than this baseline. On the other hand, the enhanced compositional approach yields a 5.1% performance improvement over the baseline, and 9% improvement over the pre-enhancement system. The improvement is observed for all factuality values (3.5% for fact, 58% for probable, 36.9% for possible, 163.4% for counterfact as well as from 0% F_1_ score to 50% for doubtful). These results should be interpreted in the light of the predominance of factual predications in the corpus as well as the inter-annotator agreement with F_1_ score (0.91), which is generally viewed as an estimate of the upper bound on machine performance [39].

**Table 7:** Evaluation results on the test set.

	Precision (%)	Recall (%)	F_1_ (%)	Accuracy (%)
*Majority baseline*				89.9
fact	89.9	100.0	94.7	
*Pre-enhancement rule-based approach* [29]				86.7
fact	95.6	91.2	93.4	
probable	29.6	79.1	43.1	
possible	37.6	67.2	48.2	
doubtful	0.0	0.0	0.0	
counterfact	34.8	22.9	27.6	
uncommitted	0.0	0.0	0.0	
*Enhanced compositional rule-based approach*				**94.5**
fact	95.6	98.8	97.2	
probable	66.7	71.6	69.1	
possible	86.8	54.1	66.7	
doubtful	100.0	33.3	50.0	
counterfact	100.0	57.1	72.7	
uncommitted	95.5	40.4	56.8	
*Supervised machine learning with features from Miwa et al*. [28]				89.8
fact	90.4	99.5	94.7	
probable	50.0	5.9	10.5	
possible	40.0	3.3	6.1	
doubtful	0.0	0.0	0.0	
counterfact	100.0	2.9	5.6	
uncommitted	20.0	3.9	6.6	
*Supervised machine learning with additional features*				92.9
fact	94.5	99.0	96.7	
probable	57.4	51.5	54.3	
possible	80.0	45.9	58.3	
doubtful	0.0	0.0	0.0	
counterfact	88.9	45.7	60.4	
uncommitted	46.7	13.7	21.2	

The system performance based on supervised machine learning is also shown in Table 7. Using meta-knowledge classification features from Miwa et al. [28] did not yield any improvement over the majority baseline, with only a handful of predications labeled as non-factual. Using the additional features proposed for classification, we were able to improve the accuracy to 92.9%, which is still lower than the accuracy achieved with the enhanced rule-based approach. For the fact class, the F_1_ scores of the two approaches were comparable; however, the rule-based approach performed significantly better for other classes. Also note that the single most important feature for the classifier was found to be the scalar modality value feature, which depends on predication composition, a core aspect of the rule-based approach.

We performed an ablation study for the enhanced compositional approach by removing each of the three enhancements discussed above and measuring the system performance. These results, presented in Table 8, show that the semantic path constraint had the largest overall impact, while enhancing negated argument processing led to a significant performance improvement in recognizing the counterfact class.

**Table 8:** Ablation study for the enhanced compositional approach.

	Precision (%)	Recall (%)	F_1_ (%)	Accuracy (%)
*Enhanced compositional rule-based approach*				**94.5**
fact	95.6	98.8	97.2	
probable	66.7	71.6	69.1	
possible	86.8	54.1	66.7	
doubtful	100.0	33.3	50.0	
counterfact	100.0	57.1	72.7	
uncommitted	95.5	40.4	56.8	
*Without pruning*				94.3
fact	95.6	98.6	97.1	
probable	66.2	70.2	68.1	
possible	86.8	54.1	66.7	
doubtful	66.7	33.3	44.4	
counterfact	90.9	57.1	70.2	
uncommitted	87.5	40.4	55.3	
*Without enhancement in negated argument processing*				94.2
fact	95.4	98.8	97.1	
probable	66.7	71.6	69.1	
possible	80.5	54.1	64.7	
doubtful	100.0	33.3	50.0	
counterfact	100.0	40.0	57.2	
uncommitted	95.5	40.4	56.8	
*Without semantic path constraint*				93.5
fact	95.9	97.7	96.8	
probable	52.8	71.6	60.8	
possible	72.9	57.4	64.2	
doubtful	100.0	33.3	50.0	
counterfact	87.5	60.0	71.2	
uncommitted	95.3	38.5	54.8	

We attempted to improve the machine learning approach in various ways. For example, to address the imbalance in the dataset, we oversampled the instances labeled with non-fact values. We also experimented with a two-stage classifier, which classified predications as fact and non-fact in the first stage and further classified the non-fact into other factuality classes. Finally, we varied the size of the training and test sets. None of these experiments yielded an improvement in performance.

In summary, for assigning factuality values to SemRep predications, improving over a trivial baseline is quite challenging, as indicated by the results obtained with the classifier that incorporates features from Miwa et al. [28]. On the other hand, a rule-based approach based on careful linguistic analysis and deeper semantic processing provides state-of-the-art performance, even though there seems to be room for improvement.

### Error analysis

We analyzed and categorized the errors made by the enhanced compositional method. The distribution of these errors is shown in Table 9. We provide examples from the most frequent three types of errors below.

**Table 9:** Distribution of error categories for the enhanced compositional method.

Category	%
Factuality triggers	29.9
Mapping rules	27.7
Argument identification	16.8
Scalar modality value composition	10.9
Preprocessing	5.8
Graph transformation	5.1
Comparative structures	2.2
Syntactic parsing	1.5

The most frequent type of error involves presence/absence of factuality triggers. A typical example is given in Example (4), in which no factuality trigger is present, leading the system to assign it the value fact. The predication is annotated as uncommitted. We experimented with a rule specific to predications extracted from titles, which stipulated that such predications be considered uncommitted, when a main verb is not present in the title sentence. While this rule yields the correct value in this example, its overall effect was negative and the rule was ultimately discarded.

(4)
  - ***Role*** *of* *kinins* *in* *chronic heart failure* *and …*
  - Kinin-ASSOCIATED_WITH-Chronic heart failure

Errors involving the mapping rules constitute the second largest class of errors. One mapping rule simply assigns the factuality value fact to all predications indicated by prepositions. There were instances in which the semantic composition yielded the correct scalar modality value, which was superseded by this mapping rule, leading to an error. In the example below, the TREATS predication is assigned the scalar modality value of 0.8_*epistemic*_, which would correctly map to probable, if not for this mapping rule. Our more fine-grained rules for predications indicated by prepositions failed to improve performance.

(5)
  - *Healthcare professionals need to consider antithrombotic and* *antihypertensive therapies* ***for*** *all* *stroke* *patients.*
  - Antihypertensive therapy-TREATS-Cerebrovascular accident

Argument identification errors were often caused by the lack of an appropriate semantic dependency type for the factuality trigger in the embedding dictionary. In Example 6, the semantic dependency that exists between the counterfactual trigger *rule out* and the predicate *cause* has the type *PREP_AS*, which is not encoded in the dictionary, leading the system to assign the value fact, instead of counterfact to this predication.

(6)
  - *…we could not rule out oral* *antidiabetic agents* *as a* ***cause*** *of* *liver disease* *…*
  - Antidiabetics-CAUSES-Liver diseases

## Conclusion

We presented an annotated corpus that focuses on factuality of semantic relations, extracted by SemRep, and experimented with two approaches, one an extension of an earlier rule-based approach and the other a machine learning approach, to assign factuality values to SemRep predications. The compositional, rule-based approach yielded better performance, although there is room for improvement. While only the factuality of SemRep predications was considered in this study, the system can accommodate any semantic relation extraction system that marks relation triggers and term mentions.

There are several limitations to the rule-based approach, which we plan to address in future work. First, the transformation rules that convert syntactic dependencies to a semantic graph are not exhaustive. It may be possible to automatically learn such transformations more generally using the recently available linguistic graphbanks for semantic parsing [40]. Second, the embedding dictionary is hand-crafted and scalar modality values encoded are based on linguistic intuitions. Automatically learning lexical items relevant to factuality and their attributes could be more robust. Recently, Lee et al. [41] presented a crowdsourcing experiment in which contributors were asked to assign factuality values to events, which were then used to calculate scalar factuality values for each event. A similar approach could potentially be applied to lexical triggers instead of events.

The resulting annotated corpus is publicly available in standoff annotation format at https://skr3.nlm.nih.gov/Factuality/. In addition, the source code for the best performing compositional approach is made publicly available as a component of the Bio-SCoRes framework (https://github.com/kilicogluh/Bio-SCoRes/) [42] and it will be incorporated into SemMedDB [15], so that researchers who exploit this repository can take advantage of the factuality feature to potentially enhance their methods. For example, rather than treating all predications equally, researchers may want to give more weight to rhetorically salient predications (claimed knowledge updates [43]), which often appear at the end of the abstract with high factuality values. While we have not evaluated them here directly, scalar modality values of predications provide an even more fine-grained representation, with numerical values, which we plan to incorporate into SemMedDB, as well.
